# BiGG: a Biochemical Genetic and Genomic knowledgebase of large scale metabolic reconstructions

**DOI:** 10.1186/1471-2105-11-213

**Published:** 2010-04-29

**Authors:** Jan Schellenberger, Junyoung O Park, Tom M Conrad, Bernhard Ø Palsson

**Affiliations:** 1 Bioinformatics Program, University of California San Diego, 9500 Gilman Dr, La Jolla, California, 92093-0419, USA; 2 Bioengineering Department, University of California San Diego, 9500 Gilman Dr, La Jolla, California, 92093-0412, USA; 3 Department of Chemistry and Biochemistry, University of California San Diego, 9500 Gilman Dr, La Jolla, California, 92093-0332, USA

## Abstract

**Background:**

Genome-scale metabolic reconstructions under the Constraint Based Reconstruction and Analysis (COBRA) framework are valuable tools for analyzing the metabolic capabilities of organisms and interpreting experimental data. As the number of such reconstructions and analysis methods increases, there is a greater need for data uniformity and ease of distribution and use.

**Description:**

We describe BiGG, a knowledgebase of Biochemically, Genetically and Genomically structured genome-scale metabolic network reconstructions. BiGG integrates several published genome-scale metabolic networks into one resource with standard nomenclature which allows components to be compared across different organisms. BiGG can be used to browse model content, visualize metabolic pathway maps, and export SBML files of the models for further analysis by external software packages. Users may follow links from BiGG to several external databases to obtain additional information on genes, proteins, reactions, metabolites and citations of interest.

**Conclusions:**

BiGG addresses a need in the systems biology community to have access to high quality curated metabolic models and reconstructions. It is freely available for academic use at http://bigg.ucsd.edu.

## Background

Metabolism is the structure and behavior of chemical reaction networks that occur in living organisms in order to maintain life. It is intrinsically linked to many other cellular functions and metabolic abnormalities are implicated as the cause of various diseases. Over the last 100 years, the list of reactions comprising an organism's metabolism has largely been catalogued. This reductionist process has focused on characterizing individual reactions in great detail. However, as the body of metabolic knowledge grew, so did the desire to integrate it into comprehensive models to simulate, predict and ultimately understand its behavior on a systems level. Kinetic models utilizing a system of differential equations are an established method of modeling biochemical pathways [1]. This field is an active area of research with an extensive number of models [2-5] as well as computational tools [6,7] available. Kinetic modeling suffers from the difficulty of requiring comprehensive knowledge of kinetic parameters to sufficiently define the system. The parameters have proven difficult to measure in a consistent fashion and are often unknown [8,9]. A consequence is that the scope of kinetic models tends to be limited.

In contrast, constraint based modeling based on genome-scale metabolic reconstructions aim to include every known reaction for an organism, through the integration of genome annotation and biochemical knowledge. Reactions are defined simply by their reaction stoichiometry, and the networks are easily converted to mathematical models on which constraint-based analysis can be applied. In this paradigm, model predictions depend on constraints through reaction fluxes and an inferred metabolic objective, rather than on precisely defined kinetic parameters. Metabolic reconstructions have proven broadly useful for a number of applications. Case studies have been reviewed in [10].

In recent years, the publication of hundreds of genomes, with various databases such as KEGG [11], Biocyc [12] and Reactome [13] describing their annotation, has simplified the task of creating drafts of genome-scale metabolic reconstructions [14,15]. This has spurred the development of an ever increasing number of reconstructions [16-22]. It is important to note that reconstructions derived directly from genome annotation may contain several gaps or incorrect annotations, leading to errors in model predictions. In order to be useful for prediction, models must undergo multiple rounds of manual curation and testing [23]. A number of widely-used manually-curated, component-by-component (bottom-up) reconstructions of genomic and bibliomic data have been published, creating the need for a systematized biochemically, genetically and genomically structured (BiGG) knowledgebase of metabolic reconstructions.

### Model Reconstruction Process

A general bottom-up metabolic reconstruction process has been formulated and detailed in [16,17]. Initially, a parts list is assembled from existing databases (most notably KEGG [11], EntrezGene [24]) giving a crude reconstruction scaffold. This reconstruction is refined through an extensive review of primary literature, review articles, textbooks, and other specialized databases. A mathematical representation (**S **matrix) of the reconstruction is created and used to validate network structure by testing functionality, such as growth under some condition or the ability to produce a specific metabolite. Furthermore, gap analysis identifies possible missing reactions by finding so called 'dead end' metabolites which can be produced by the network but not consumed. Failure of network validation tests and the existence of gaps suggest targeted literature searches or experiments, which can be used to improve the model. Each reaction is verified individually and a confidence score can be assigned by the curator. A model may undergo several iterative rounds of validation and changes before it reaches a satisfactory state and is published, a process which can take up to a year of time. Because of the great effort involved, there have been attempts to partially automate the process [25-31] and split work through collaboration [32].

### Gene-Protein-Reaction associations

**Figure 1:**
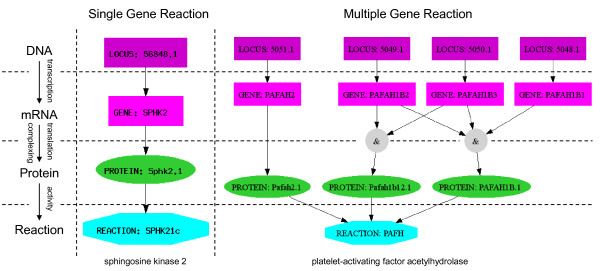
**Gene Protein Reaction interactions**. Gene Protein Reactions formulas for two Human Recon1 reactions. Each graph indicates the relationship between genes (purple), transcripts (magenta), protein (green), and reaction (teal). A) Sphingosine kinase 2 (SPHK21c) is associated with only one gene. B) Platelet-activating factor acetylhydrolase (PAFH) can be transcribed by either gene *PAFAH2 *or in combination of genes *PAFAH1B1*, *PAFAH1B2*, and *PAFAH1B3*. The GPR expression for this reaction is (5051.1) or (5049.1 and 5050.1) or (5049.1 and 5050.1 and 5048.1).

Most biological reactions require enzymatic catalysis to occur. Thus the 'on' or 'off' state of each reaction in the network is controlled by the genotype and expression level of associated genes. In the simplest case, a reaction is catalyzed by only a single enzyme which is coded for by a single gene. The expression and translation of that gene implies the feasibility of the reaction, and vice versa. More complex cases involve multiple genes and proteins whose relationship is described using Boolean logic. A single protein may be composed of subunits coded by two (or more) genes. If all of these subunits are required for the catalytic activity of the protein, the activity is modeled as an 'and' logic ('gene A and gene B'). Alternatively, the model allows for equivalent proteins (isozymes) to catalyze the same reaction. In this case, the presence of either protein is sufficient to establish the activity of the reaction and an 'or' logic is used ('protein A or protein B'). Other phenomena, which are representable in the Boolean framework, are alternative splicing ('or' logic) and obligate protein complexes ('and' logic). Collectively, these Boolean logic statements relating genes, proteins, and reactions are named GPRs. If a GPR statement of a reaction evaluates to 'true,' then its corresponding reaction is said to be feasible. Thus, GPRs may be used to evaluate the effects of gene knockouts and gene regulation on the metabolic reconstructions, ruling out reactions whose genes are not available. GPRs may also be displayed graphically. Figure 1 shows two of the possible GPR associations found in BiGG.

## Construction and content

### Reconstructions

BiGG is currently capable of browsing and exporting the contents of seven different genome-scale reconstructions of six organisms (see Additional File 1): *Homo sapiens *Recon 1 [33], *Escherichia coli i*JR 904 [34] and *i*AF1260 [35], *Saccharomyces cerevisiae i*ND750 [36], *Staphylococcus aureus i*SB619 [37], *Methanosarcina barkeri i*AF692 [38] and *Helicobacter pylori i*IT341 [39]. These reconstructions span all three major branches of the tree of life and include two model organisms.

A global reconstruction of the human metabolic network, *H. sapiens *Recon 1, was recently completed [33]. The initial human reconstruction was based on gene information from the KEGG, EntrezGene, and H-Invitational [40] databases and was curated by evaluation of primary literature, reviews, and textbooks. Recon 1 represents a valuable tool as a scaffold for analysis of "-omics" data sets.

A variety of microorganisms have also been reconstructed. The *E. coli *reconstructions, *i*JR904 and more recently *i*AF1260, are the most complete and most used of these reconstructions. *i*JR904 has been used for the prediction of adaptive evolution endpoints [41] and the engineering of lactate producing *E. coli *strains [42]. *H. pylori*, another Gram-negative enterobacteria that lives in the human stomach and has been shown to cause ulcers and gastritis, has a reconstruction, *i*IT341. *i*AF692 is a reconstruction for the methanogenic archaebacteria *M. barkeri*. *i*SB619 is a reconstruction of the infectious Gram-positive bacteria *S. aureus *of interest due to high rates of infection and increasing resistance to antibiotics. As more reconstructions are published, they will be added to BiGG.

**Figure 2:**
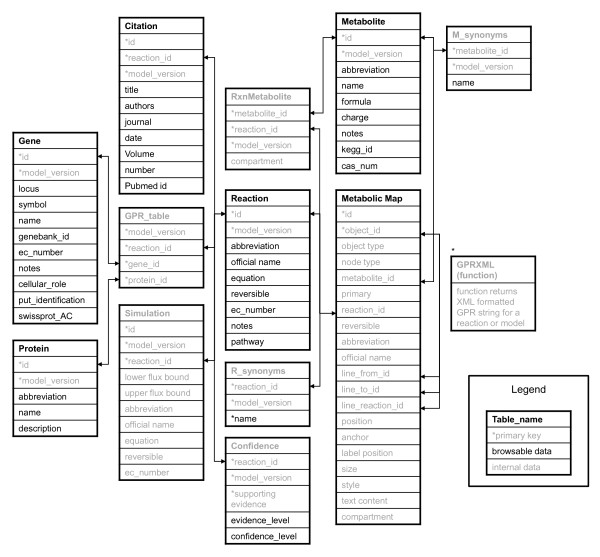
**Database Schema**. BiGG is hosted on a Simpheny™ server running an Oracle database. Starred columns indicate primary keys. Arrows indicate foreign key relationships. GPR_table stores the relationship between reactions, proteins, and genes. All tables and entries shown in black are directly viewable by the user. Grey entries are used internally only. GPRXML (marked by *) is a function which returns the XML formatted GPR string given a reaction ID.

All reconstructions in BiGG were developed on the Genomatica Simpheny™ platform. This system includes quality control features to track genes, proteins and reactions, as well as simulation tools to computationally validate models. The models are built from a shared universal database of compounds and reactions. It is therefore not possible to incorporate reconstructions developed with other tools. The reconstructions are stored on a Genomatica (San Diego, CA) supplied server running an Oracle™ database. Access to this database is provided by a read-only client with several tables and views for accessing information on Reactions, Metabolites, Genes, Proteins, Maps and Citations (Figure 2). All queries are performed by a Linux/Apache/Perl Server using the CGI and DBI modules.

### Browsing

The two main functions of BiGG are browsing content and exporting whole reconstructions. The browser is designed for querying the content and comparing different reconstructions whereas the exporter is primarily designed to enable further computational analysis by other software packages.

The BiGG browser contains entries for metabolic reactions, metabolites, genes, proteins, and literature citations (Figure 2). Reaction entries contain information such as the balanced equation, compartment localization, EC number [43], reversibility, author comments, and links to references. Metabolite entries contain information such as chemical formula and charge under physiological conditions. The GPR relationships are displayed as text or graphs using the graphviz package http://graphviz.org. Hyperlinks to other databases are included whenever provided by the authors of the reconstructions. These include NCBI Entrez gene database [44], Uniprot/Swissprot [45] for genes, and KEGG and CAS http://www.cas.org identifications for metabolites.

Reactions and metabolites can be searched through the Search Reactions and Search Metabolites pages. Reactions may be searched for by name, EC number, or associated gene. Alternatively, all reactions in a model may be listed by using the model name as the only search parameter. It is also possible to specify compartment, pathway, or metabolite participation. Results may be limited by only including reactions with known gene associations, high or low confidence, or by excluding transport reactions. In addition, reactions may be searched across reconstructions allowing for model comparison. Lists of reactions matching a set of criteria may be exported as a tab delimited flat file. The exported files can contain information for multiple models, simplifying model comparison.

**Figure 3:**
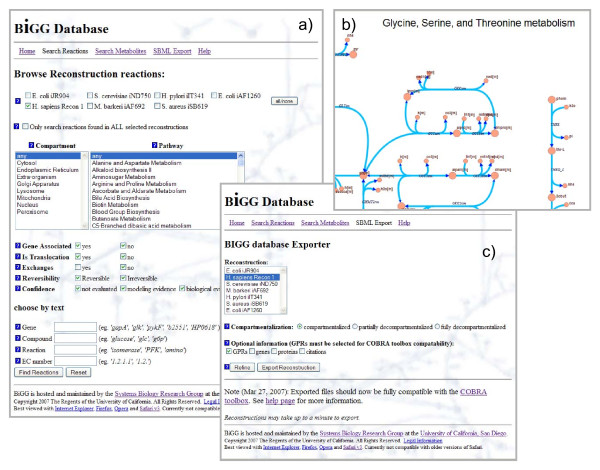
**The BiGG website**. The BiGG knowledgebase can be accessed through a web browser. It has been tested with Mozilla Firefox, Microsoft Internet Explorer, Opera, and Safari. Three screenshots in Firefox show: (a) the content browser, (b) map viewer, and (c) the export tool.

Metabolites may be searched for by name, KEGG ID, CAS ID, or charge. Just as for reactions, limiting searches by compartment, pathway, and organism is possible. In addition to basic metabolite information such as formula and charge, lists of reactions in which the metabolite participates are listed and categorized by the metabolite's role as a reactant or a product. Lists of metabolites matching a set of search criteria may be exported as tab delimited flat file, and contain information such as metabolite name, abbreviation, formula, KEGG ID, and CAS ID. The browser interface is shown in Figure 3a.

### Maps

For visualization, curated metabolic maps are provided for many organisms in BiGG. These maps show metabolites, reactions, and text markup. Some large reconstructions (in particular human Recon 1) have several maps for different metabolic subsystems. Maps are rendered with Scalable Vector Graphics (SVG) for smooth scaling and are hyperlinked back to the entries in the database. A small portion of a metabolic map from the human reconstruction is shown in Figure 3b.

### Exporting

The second function of the BiGG knowledgebase is exporting reconstructions as Systems Biology Markup Language (SBML) files [46], which are specifically designed to work with the Matlab COBRA toolbox [47] and Systems Biology Research Tool [48] for performing flux balance analysis and other computations. This XML format is widely used for distributing systems biology models [46]. By default, only entries for compartments, metabolites (the <species> tag), and reactions are included. The user has several options available to customize export as detailed below (see Figure 3c for interface).

#### Decompartmentalization

A reconstruction may be exported as a decompartmentalized model. A compartment in a metabolic reconstruction is a distinct pool of metabolites and their reactions. Metabolites are exchanged among compartments by transporter reactions. All reconstructions included in BiGG have at least two compartments: Cytosol and Extra-organism. Additionally, reconstructions of eukaryotic organisms have internal compartmentalization modeling subcellular organelles. A *partially decompartmentalized *reconstruction removes these internal compartments and relocates their reactions and metabolites to the Cytosol. A *full decompartmentalization *removes internal compartments as well as the boundary between the Extraorganism and Cytosol compartments, creating a single-compartment system. In either case, internal transporters are removed.

It should be noted that the utility of decompartmentalization lies in model comparison rather than a basis for simulations. For instance, reactions such as ATP synthase are driven by an electrochemical gradient across compartment boundaries. In the decompartmentalized model there can be no gradients thus the ATP Synthase reaction becomes thermodynamically incorrect, creating unpredictable outcomes with some optimizations. As a rule, decompartmentalization should only be used for comparative purposes. Computational studies should only be performed on the full models.

#### Associated Genes, Proteins, and Citations

The exported SBML file may include information on genes, proteins and citations. Because the SBML specification does not include fields for this kind of data, this information is stored in the 'notes' field of the reaction entries.

#### GPR statements

The notes field of the Reaction entries in the exported SBML file can include Boolean strings corresponding to the GPR statements. The GPR field is read and interpreted by the COBRA toolbox but should be ignored by other programs.

#### From Reconstructions to Models

Reconstructions are the basis for computational models. The process of converting a reconstruction into a model is performed by the curator and is reviewed in [16,49]. Upper and lower bounds are placed on reaction rates, bounding the space of flux distributions. An objective function is added, often corresponding to the biomass production. The reconstruction, bounds and objective function together comprise the model exported by BiGG.

Most of the simulations are run by default under parameters simulating aerobic growth condition in glucose minimal medium. This is modeled by the constraints on fluxes of the model's exchange reactions. For instance, modeling of an aerobic environment with glucose minimal media must allow for glucose, oxygen, ammonium ion, salts, and other ions to be up-taken but other carbon sources only excreted. These bounds are included in the SBML file along with the objective coefficients of each reaction and flux distribution. For simulating other conditions there is a web based interface for changing the bounds on any reaction by pressing the "refine" button. In this way, SBML files corresponding to different media compositions can be created.

#### Compatibility

SBML files conform to the level 2 version 1 specification and are compatible with the COBRA toolbox [47] which contains many computational procedures. Using the COBRA toolbox, the SBML file exported from BiGG may be imported as a network data structure into Matlab. This structure includes the stoichiometric matrix, gene and reaction information, and GPR associations. The toolbox then allows the user to interrogate the model's solution space using a variety of tools, including flux balance and flux variability analysis, random sampling, and robustness and gene deletion analysis. Matlab scripting can be used to combine methods or develop new methods not provided in the toolbox. The JAVA based Systems Biology Research tool [48] is another software package tested to work with the SBML files exported from BiGG.

## Utility

### Querying General Statistics of Reconstructions

**Figure 4:**
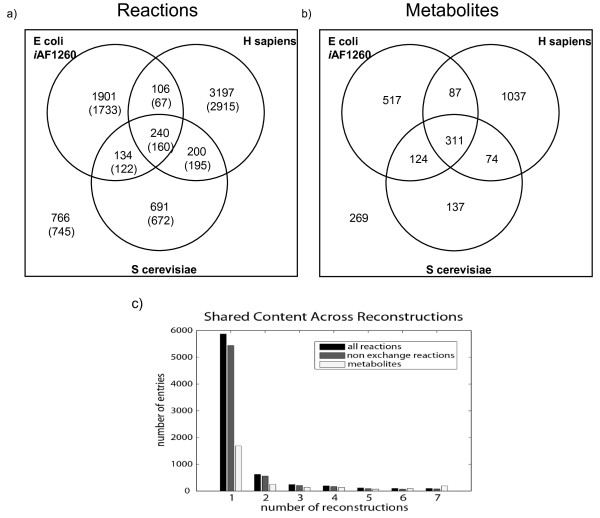
**Content of BiGG**. The three largest reconstructions in BiGG are *E. coli i*AF1260, *S. cerevisiae i*ND750, and *H. sapiens *Recon 1. Their shared content can be queried with BiGG: a) The shared reactions. Non-exchange reactions are shown in parenthesis. b) The number of shared metabolites. c) The distribution of reactions and metabolites across all seven reconstructions.

The capability of BiGG to browse and compare multiple reconstructions was used to provide a comparison of the available reconstructions. The seven metabolic reconstructions available via BiGG vary in size from 551 reactions in *H. pylori*, to 3743 reactions in the human reconstruction. The total number of metabolites in BiGG is 2556, of which more than half (1509) are found in the Human Reconstruction (Additional File 1). A set of 96 core reactions is shared by all reconstructions, while most reactions were found in only one reconstruction (Figure 4c). Ubiquitous reactions include those involved in central metabolism, nucleotide and amino acid metabolism, and several exchange reactions (results not shown). Translocation reactions tend to be unique to particular reconstructions. The three largest reconstructions (*H. sapiens*, *E. coli*, *S. cerevisiae*) share a total of 240 reactions, 80 of which are exchange reactions (Figure 4a).

The content distribution usage in BiGG is shown in Figure 4C. Most reactions are only found in one reconstruction although 1167 are shared between at least two. Metabolites are shared more frequently. A smaller fraction of metabolites is unique to just one reconstruction (Figure 4b, c).

### Case Study - Orphan reactions

All reconstructions have knowledge gaps where information on components is not available. One example is orphan reactions which are reactions for which the catalyzing enzyme is unknown. The BiGG knowledgebase can be used to study and help fill in these knowledge gaps by 1) listing all orphan reactions and 2) displaying any other reconstruction that use these reactions. *E. coli *metabolism has been studied extensively and most of the predicted open reading frames have at least putative functional assignments. The *E. coli *metabolic network reconstruction has gone through several iterations and has become more complete [35]. The iJR904 reconstruction contains 58 orphan reactions (Additional File 2). Six are labeled spontaneous, meaning they can proceed without the aid of an enzyme and thus do not require an associated gene. A further reaction is the 'ATP maintenance requirement' which is a virtual reaction representing the turnover of ATP to ADP to maintain cellular functions. A total of seven reactions were removed in *i*AF1260, including two 'lumped' reactions (reactions which are stoichiometric representations of more complex processes) which are also not gene associated. These two have been replaced with elementary reactions. This leaves 44 reactions with missing gene associations in *i*JR904. Fourteen now have genes in *i*AF1260 while the remaining 30 do not. Twelve of these 30 are found in at least one other reconstruction, forming the basis for further searching. Analyses like these provide an overview of the state of reconstructions and can pinpoint areas of future focus. Performing this analysis without the BiGG knowledgebase would be possible although cumbersome.

### Future Development

#### Additional reconstructions

Two notable reconstructions in development are *Bacillus subtilis *and *Haemophilus influenzae*. As they become available, they will be added to BiGG as well. Currently, only *E. coli *has more than one reconstruction version, but in the future, we plan on hosting different (older) versions of other reconstructions as well. Currently, it is only possible to host reconstructions created within the Simpheny software and at the moment there is no way to import other groups' reconstructions. This may change in the future.

#### Downloadable Maps

The BiGG knowledgebase is designed to work with the COBRA toolbox. Version 2.0 of this toolbox will be released soon and will include a visualization component. The BiGG maps will be downloadable in a custom text format containing coordinates of all metabolites and reaction control points. This is imported into COBRA and displayed in a customizable fashion. Colors and sizes can be changed on a per-reaction basis to visualize biological results.

#### Pre-made constraints/Media

At present, exported models contain one set of lower and upper flux bounds. Lower bounds of irreversible reactions are automatically set to 0 and upper bounds are either set to arbitrarily large values (eg. 999999) or physiologically determined rates. However, to run meaningful simulations, the bounds of the exchange fluxes must be specified to match the environment. For instance, modeling of an aerobic environment with glucose minimal media must allow for glucose, oxygen, ammonium ion, and salts to be taken up, but not other extracellular species. Currently, this must be done manually via the export "refine" button, but in the future, libraries of bounds (constraint) vectors will be added to the SBML files to allow the user to specify media conditions.

## Discussion and Conclusions

The reconstructions and models in BiGG have several specific features necessary to compute within the COBRA framework: 1) Each reconstruction in BiGG is manually curated. Exotic transformations unique to an organism may be absent from databases and must be pulled from primary literature. 2) BiGG uses both genetics and literature based data to assess whether a reaction is present. If the genetic basis for a reaction is unknown but the reaction is described in the literature, it will be included without associated genes (an "orphan reaction"). 3) The curators of BiGG reconstructions have the option of providing confidence levels for reactions which can be used when evaluating resultant models. These levels, along with reaction notes, provide an assessment of the confidence that a reaction is correctly included in the model. 4) Boolean relationships between genes, proteins and reactions (GPRs) are described in BiGG. This information is necessary for the proper modeling of mutations or gene knockouts. 5) All reactions in BiGG are mass and charge balanced. In some metabolic databases, simple species such as H^+ ^and H_2_O are simply ignored [50]. Failure to balance reactions can result in unrealistic metabolic predictions. 6) Compartmentalization in BiGG gives an accurate description of reactions involving membrane transporters. This is required for simulation of gradient driven pumps [35]. 7) BiGG bridges the gap between a reconstruction and a model. The exported SBML files have all been validated and can be used to make predictions about growth rate, predicting the effect of gene deletions (MOMA [51]), and other COBRA framework methods. Taken together, these 7 features allow BiGG to represent metabolic reconstructions and the underlying chemistry in an accurate way. While individually these features are not unique to BiGG, no other resource including all of these features. The content of other genome-scale metabolic databases cannot be used directly for modeling in the COBRA framework [18]. The advent of genome sequencing has led to an explosion of systems biology methods which attempt to study properties of whole networks rather than individual parts. The results (often referred to as 'emergent properties') cannot be explained by studying the individual parts separately. Due to the scale of the models used, they are quite time consuming to develop and it is beneficial to share them with other researchers. The BiGG knowledgebase provides the first collection of curated, high quality metabolic reconstructions suitable for study with COBRA methods. We expect it to continue to be a useful resource in the future as new and updated models are added to the database.

## Availability and Requirements

The BiGG knowledgebase is available online at http://bigg.ucsd.edu/. A JavaScript enabled browser is required for browsing and exporting. The map viewer requires SVG support. BiGG data and results require a password which is made freely available for academic use.

## Abbreviations

BiGG: Biochemically, Genetically and Genomically structured; CAS: Chemical Abstracts Service; COBRA: Constraint Based Reconstruction and Analysis; GPR: Gene, Protein, Reactions; KEGG: Kyoto Encyclopedia of Genes and Genomes; SBML: Systems Biology Markup Language.

## Authors' contributions

JS built the database interface and content browser. TC and JP wrote the export tool and GPR visualization. JP wrote the map visualization. JS and TC drafted the manuscript. All authors read and approved the final manuscript.

## Supplementary Material

Additional file 1 **Table 1 - Contents of BiGG**. BiGG currently contains 7 reconstructions including two versions of *E. coli*. There are a total of 7234 unique reactions and exchanges in the database. Exchange reactions carry metabolites from the extracellular 'compartment' across the system boundary and are not technically part of the metabolic reconstruction. Translocation reactions carry a metabolite between compartments (possibly performing other transformations). Reactions can be gene associated or not. Every reconstruction contains the 'cytosol' and 'extracellular' compartment. Human and yeast contain 'endoplasmic reticulum', 'mitochondria', 'peroxisome', 'nucleus'. The 'periplasm' in *i*AF1260, vacuole in yeast, and lysosome in human are unique to these reconstructions. Click here for file

Additional file 2 **Table 2 - Orphan reactions**. 58 *E. coli i*JR904 orphan (non-gene associated) reactions are categorized by current status. 'Found in 1260' shows whether the reactions are found in reconstruction iAF1260. 'Y*' indicates that the reaction is found in iAF1260 in modified form (usually because of the addition of the periplasm compartment). Notes: spont. -spontaneous, lump - lumped reaction, virtual - virtual reaction, P - *H. pylori*, S - *S. aureus*, Y - *S. cerevisiae*, H - *H. sapiens*, Click here for file
